# Supplementary figures and images for: ASAP-SML: An antibody sequence analysis pipeline using statistical testing and machine learning
Source: PLoS Comput Biol. 2020 Apr 27;16(4):e1007779. doi: 10.1371/journal.pcbi.1007779 (PMC7205315; doi:10.1371/journal.pcbi.1007779)

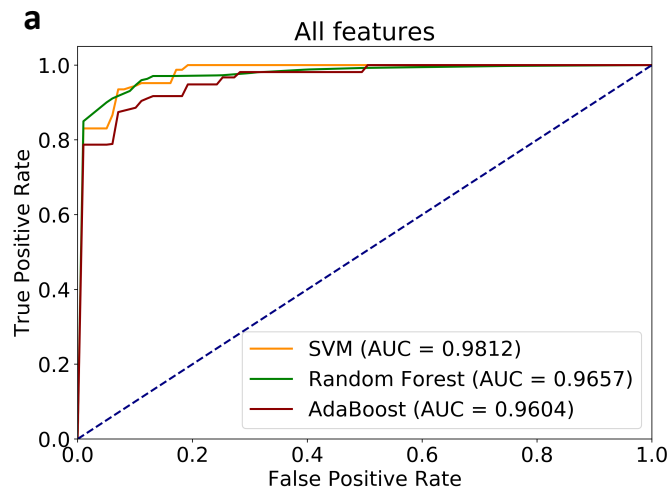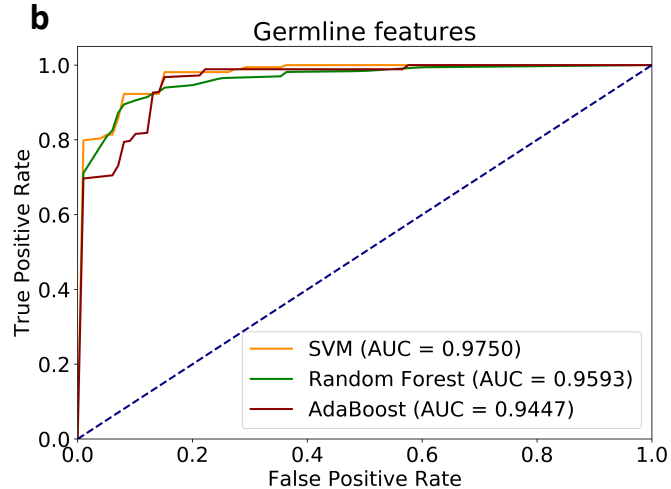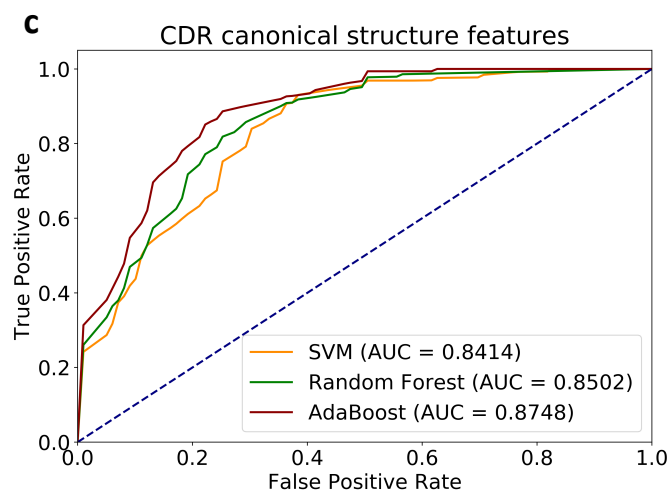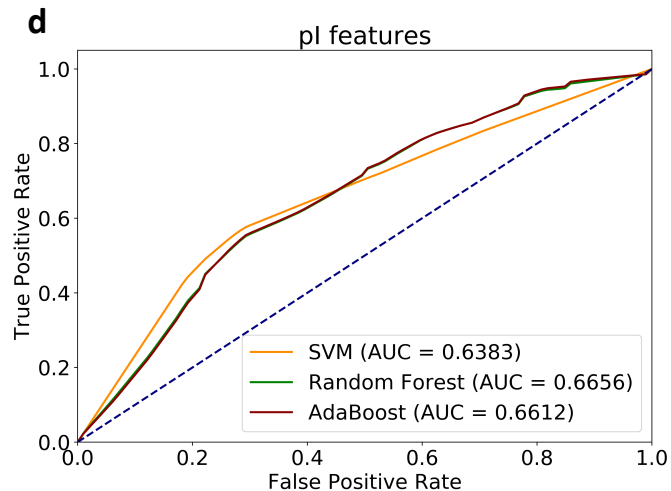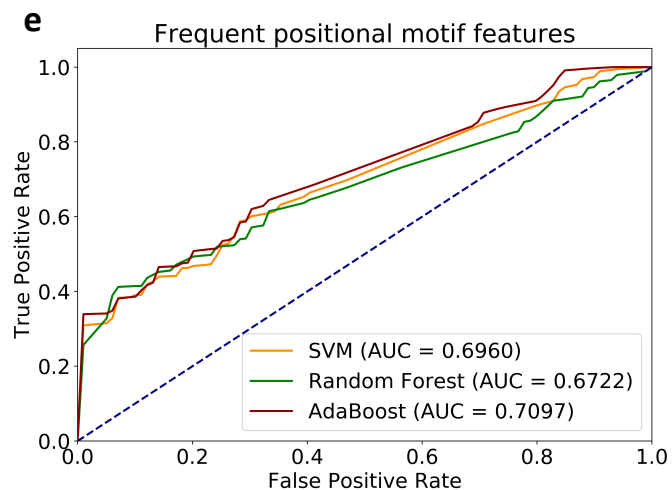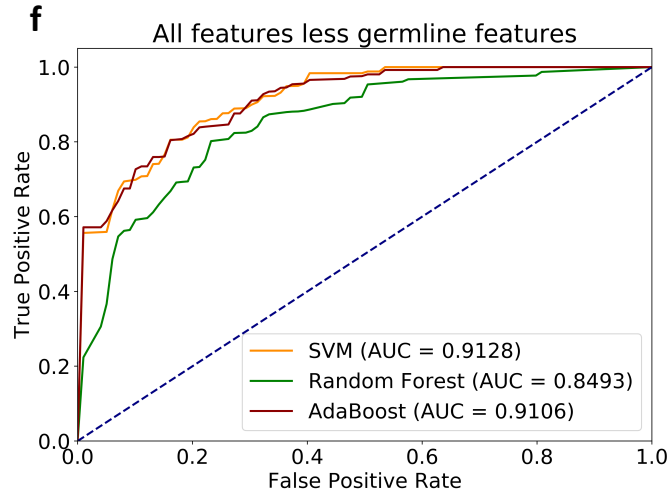

Supplement: S1 Fig — AUC for classification using SVM, random forest AdaBoost algorithms when using (a) all features, (b) only germline features, (c) only CDR canonical structure features, (d) only pI features, and (e) only frequent positional motif features, (f) all features excluding germline features. (PDF) [file pcbi.1007779.s001.pdf]

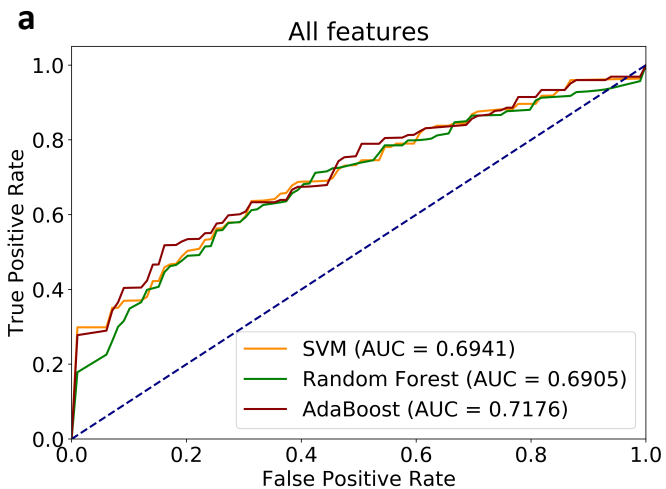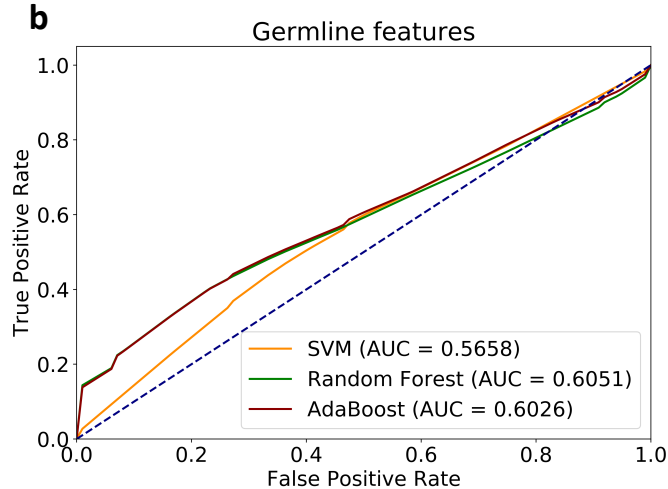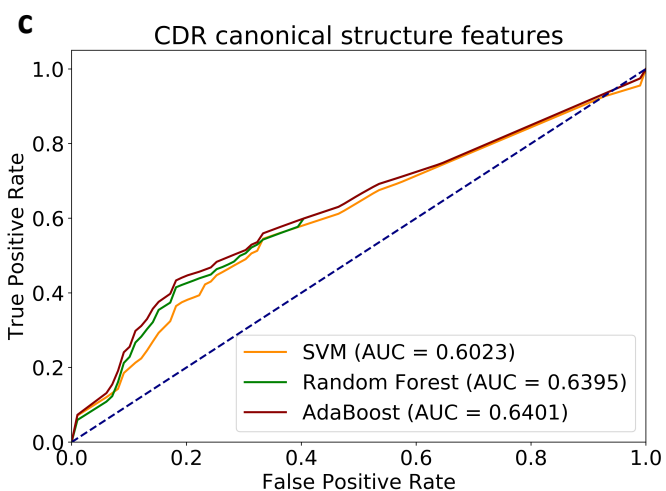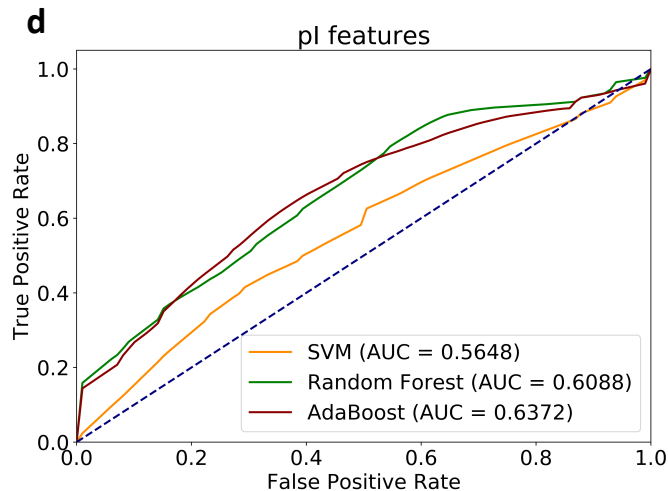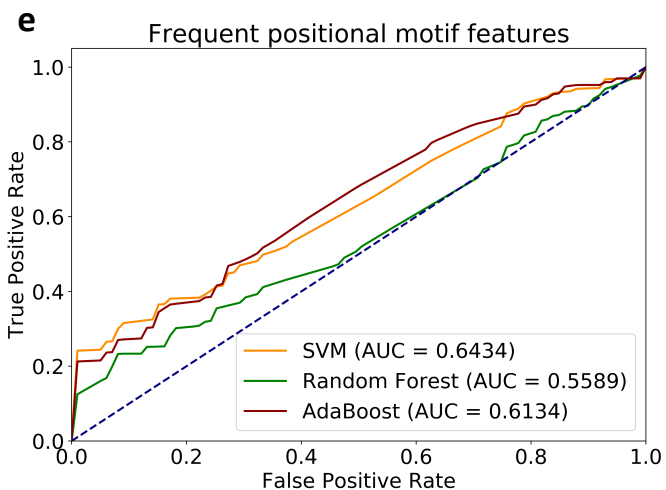

Supplement: S2 Fig — AUC data is reported for classification when using (a) all features, (b) only germline features, (c) only CDR canonical structure features, (d) only pI features, and (e) only frequent positional motif features. (PDF) [file pcbi.1007779.s002.pdf]

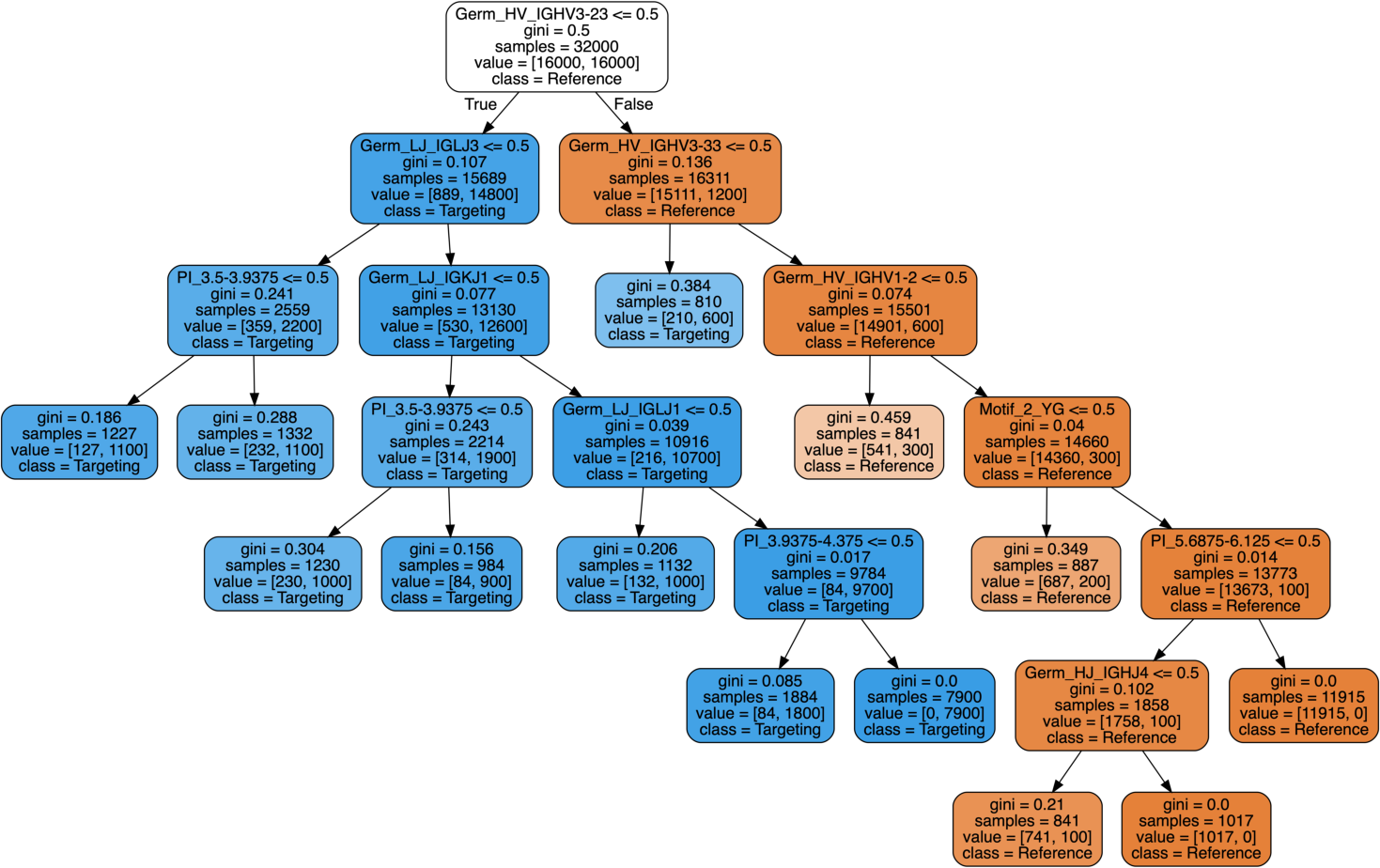

Supplement: S3 Fig — With a desired dataset size of 160, which is the size of representative MMP-targeting sequences, and k = 100 sampling iterations, each set had 160*100 sequences. The label within each node reflects the following: the feature value, the Gini impurity score, the number of samples within the tree rooted at that node, a value providing a listing of the number of samples that are from the reference set followed by the number of samples that are from the MMP-targeting set, and a node classification label indicating if the node is dominated by reference or MMP-targeting sequences. (TIF) [file pcbi.1007779.s003.tif]
